# A Stepped-Spacer FinFET Design for Enhanced Device Performance in FPGA Applications

**DOI:** 10.3390/mi16080867

**Published:** 2025-07-27

**Authors:** Meysam Zareiee, Mahsa Mehrad, Abdulkarim Tawfik

**Affiliations:** 1 Department of Design, Manufacturing and Engineering Management, University of Strathclyde, Glasgow G1 1XQ, UK; 2 Faculty of Technology, School of Electrical and Mechanical Engineering, University of Portsmouth, Portsmouth PO1 3DJ, UK

**Keywords:** S^3^-FinFET, SOI FinFET, FPGA, spacer engineering

## Abstract

As transistor dimensions continue to scale below 10 nm, traditional MOSFET architectures face increasing limitations from short-channel effects, gate leakage, and variability. FinFETs, especially junctionless FinFETs on silicon-on-insulator (SOI) substrates, offer improved electrostatic control and simplified fabrication, making them attractive for deeply scaled nodes. In this work, we propose a novel Stepped-Spacer Structured FinFET (S^3^-FinFET) that incorporates a three-layer HfO_2_/Si_3_N_4_/HfO_2_ spacer configuration designed to enhance electrostatics and suppress parasitic effects. Using 2D TCAD simulations, the S^3^-FinFET is evaluated in terms of key performance metrics, including transfer/output characteristics, ON/OFF current ratio, subthreshold swing (SS), drain-induced barrier lowering (DIBL), gate capacitance, and cut-off frequency. The results show significant improvements in leakage control and high-frequency behavior. These enhancements make the S^3^-FinFET particularly well-suited for Field-Programmable Gate Arrays (FPGAs), where power efficiency, speed, and signal integrity are critical to performance in reconfigurable logic environments.

## 1. Introduction

As semiconductor technology continues its push into the sub-10 nm regime, conventional bulk MOSFET architectures are increasingly limited by short-channel effects, high leakage currents, and variability induced by process scaling [1,2,3]. To address these challenges, FinFETs built on silicon-on-insulator (SOI) substrates have become a widely adopted alternative due to their excellent electrostatic control, lower off-state leakage, and scalability [4,5]. Among these, junctionless FinFETs, where the source, drain, and channel are uniformly doped, offer added benefits of fabrication simplicity and reduced variability, making them suitable for ultra-scaled nodes [6].

Field-Programmable Gate Arrays (FPGAs), known for their flexibility and reconfigurability, are particularly sensitive to device-level characteristics such as leakage power, switching delay, and gate capacitance [7]. Traditional CMOS-based FPGAs face scaling bottlenecks due to increasing static power and degraded signal integrity [8]. Several studies have explored the use of FinFETs to improve FPGA performance [9,10]. For instance, prior work has demonstrated that FinFET-based FPGA fabrics can achieve significant reductions in power consumption and delay compared to planar CMOS, owing to superior channel control and reduced leakage [11]. Furthermore, FinFETs allow for multi-threshold tuning and better trade-offs between performance and power, which are key requirements in modern FPGA design [12].

However, as FinFET gate lengths shrink below 10 nm, new challenges arise. Even SOI-based and junctionless FinFETs suffer from increased gate leakage, drain-induced barrier lowering (DIBL), and subthreshold degradation, especially due to fringing fields from the drain and the gate spacers. To improve FinFET performance and mitigate these effects, several studies have explored the impact of gate spacer engineering. Sachid et al. investigated high-κ spacers, demonstrating improved drive current and reduced short-channel effects due to enhanced electrostatic control, albeit with increased parasitic capacitance [13]. Studies on wavy FinFET structures have compared various materials such as SiO_2_, Si_3_N_4_, and HfO_2_, finding that HfO_2_ spacers deliver a significant leakage reduction and improved I_ON_/I_OFF_ ratio [14]. Moreover, hybrid spacer stacks combining low-κ and high-κ dielectrics have been applied in DG-TFET and planar transistors, where SiO_2_/HfO_2_ configurations yield optimal tunneling efficiency and retention in memory-type devices [15]. Also, asymmetric spacer configurations, such as dual-κ or corner spacers, have been proposed to tailor source/drain fringe fields independently, resulting in enhanced capacitance characteristics and inverter responses in TFETs and negative-capacitance FinFETs [16].

Building upon these spacer engineering strategies, this work introduces a novel FinFET architecture, termed the S^3^-FinFET (Stepped-Spacer Structured FinFET), which employs a three-layer spacer stack composed of HfO_2_/Si_3_N_4_/HfO_2_ arranged in a stepped vertical configuration. This design not only enhances fringe field coupling near the gate, but also screens unwanted electric field penetration near the source/drain terminals, thus improving both ON- and OFF-state behavior. Through rigorous 2D TCAD simulations using the Silvaco Atlas platform, the S^3^-FinFET is evaluated for key performance metrics including transfer and output characteristics, subthreshold swing, DIBL, ON/OFF current ratio, gate capacitance profiles, and cut-off frequency.

Despite the advantages mentioned above, integrating FinFET technology into FPGA fabrics remains a nontrivial task. The performance of FPGAs is heavily dependent on the behavior of their constituent devices under static conditions, particularly in routing paths and configuration logic, where leakage and capacitance directly impact power and speed. Moreover, as gate lengths scale below 10 nm, even advanced FinFET structures encounter worsening electrostatic integrity, fringing field effects, and layout-induced variability. These challenges underscore the need for novel device architectures that specifically address FPGA-centric requirements.

The study of the S^3^-FinFET design reported here highlights its strong potential for FPGA applications. Its excellent electrostatic gate control makes it a promising candidate for integration into next-generation FPGA fabrics, enabling energy-efficient, high-speed reconfigurable systems at deeply scaled technology nodes.

## 2. Device Architecture and Simulation Framework

**Figure 1:**
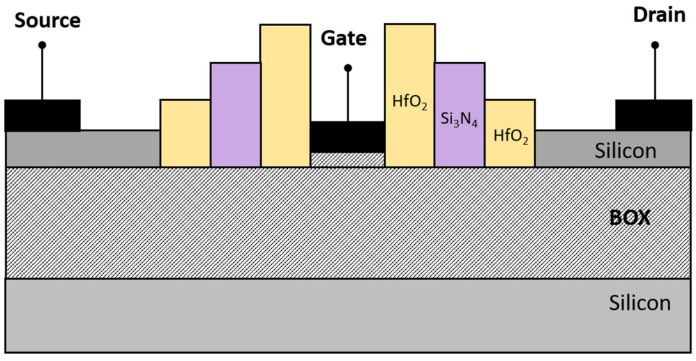
The proposed S^3^-FinFET (Stepped-Spacer Structured FinFET) structure.

The S^3^-FinFET investigated in this work is simulated using 2D Silvaco TCAD simulator [17]. The device features a gate length of 5 nm and is designed on an SOI platform, which includes an 80 nm buried oxide layer for substrate isolation, as shown in Figure 1. A high work-function metal gate (4.8 eV) is placed atop a SiO_2_ gate dielectric, with the oxide thickness set to an equivalent oxide thickness (EOT) that meets advanced technology node requirements [18].

**Figure 2:**
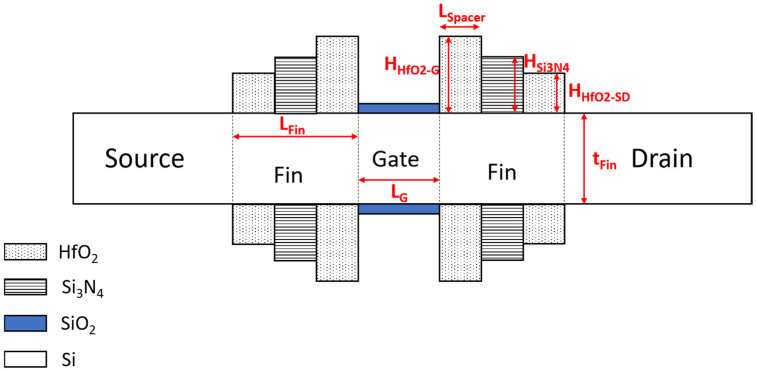
Top view of the proposed S^3^-FinFET transistor.

Unlike in traditional designs, the source, drain, and channel regions are doped entirely and uniformly to a concentration of 1 × 10^19^ cm^−3^, ensuring junctionless conduction. Both source/drain and spacer lengths are fixed at 12 nm, as shown in the top view of the proposed structure in Figure 2. The key differentiator in this structure is the use of a three-layer spacer stack. An inner HfO_2_ layer adjacent to the gate enhances gate-to-channel coupling; a middle Si_3_N_4_ layer acts as a dielectric buffer; and a second outer HfO_2_ layer near the source/drain junctions further screens fringing fields. These spacers are arranged in a stepped structure, with the HfO_2_ spacer near the gate having the greatest height, which gradually decreases toward the source and drain sides.

The simulations incorporate advanced physical models to capture quantum and high-doping effects. Carrier transport is modeled using the drift–diffusion approach, enhanced with Fermi–Dirac statistics. The quantum density-gradient model accounts for confinement and tunneling, while bandgap narrowing and band-to-band tunneling models address heavy doping and leakage. Mobility degradation from interface scattering and phonon interactions is considered via the Lombardi model, and carrier recombination is modeled using the Shockley–Read–Hall (SRH) approach.

Simulation results demonstrate that the proposed S^3^-FinFET exhibits improved performance due to the use of the three-layer spacer structure. For recombination, the Shockley–Read–Hall (SRH) model is used, which calculates the recombination rate based on carrier lifetimes and trap-assisted transitions. The SRH rate is formulated using the following formula [17]:

(1) $$R_{SHR}=\frac{np-n_{i,eff}^{2}}{\tau_{p}\left(n+n_{1}\right)+\tau_{n}(p+p_{1})}$$

where n and *p* are the electron and hole concentrations, n_i,eff_ is the effective intrinsic carrier concentration, τ_n_ and τ_p_ are the carrier lifetimes, and n_1_ and p_1_ are the trap densities.

To simplify the fabrication process, the lengths of all three spacers are kept the same. The fabrication of the proposed S^3^-FinFET begins with the formation of a silicon-on-insulator (SOI) substrate, followed by thinning of the top silicon layer and fin patterning using lithography and anisotropic etching. A gate oxide is then deposited using chemical vapor deposition (CVD), and a metal gate is formed. Subsequently, a tri-layer spacer structure is created. An inner HfO_2_ spacer is deposited and etched first, followed by the deposition and patterning of a middle Si_3_N_4_ spacer; finally, the outer HfO_2_ spacer is formed after source/drain extension implantation. Ion implantation is performed for the source/drain extensions, using a photoresist mask to protect the gate and spacer stack [19]. Afterward, selective epitaxial growth is used to form the heavily doped source and drain regions, followed by high-temperature annealing to activate the dopants. The process concludes with metallization to establish electrical contacts, completing the device structure.

Additional device parameters are provided in Table 1.

## 3. Results and Discussion

The transfer characteristics of the proposed S^3^-FinFET, compared to two other FinFET structures, a three-spacer FinFET without a stepped design and a conventional FinFET using only Si_3_N_4_ as a spacer, demonstrate the effectiveness of the novel stepped high-k spacer configuration. This design enhances fringe field penetration into the channel, thereby improving gate control across the entire fin body, which is especially beneficial at ultra-short gate lengths. The inclusion of an inner HfO_2_ layer with high permittivity extends the gate’s electrostatic reach without physically increasing gate length, improving barrier formation and reducing channel length modulation.

**Figure 3:**
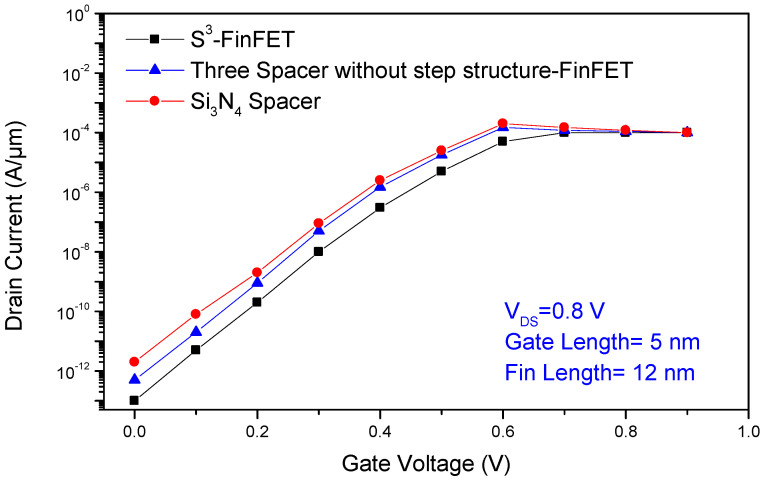
Transfer characteristics (I_D_–V_GS_) of the proposed S^3^-FinFET compared to a non-stepped three-spacer FinFET and a conventional FinFET with a single Si_3_N_4_ spacer.

As shown in Figure 3, the transfer characteristics indicate that the S^3^-FinFET achieves lower sub-threshold leakage current due to a higher vertical electric field in the OFF state, while maintaining unaffected drive current in the ON state owing to a near-zero lateral electric field. Furthermore, the subthreshold swing (SS) of the S^3^-FinFET is significantly improved compared to the other two structures, highlighting its superior suppression of thermionic leakage and excellent interface control.

**Figure 4:**
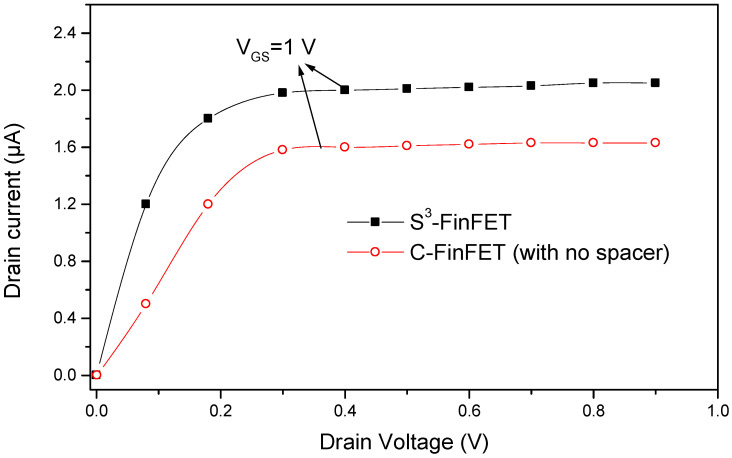
Output characteristics of S^3^-FinFET and conventional FinFET.

To further validate the performance enhancements introduced by the proposed HfO_2_/Si_3_N_4_/HfO_2_ stepped spacer structure, a comparative analysis was conducted between the S^3^-FinFET and a conventional FinFET (C-FinFET) without any spacer. Figure 4 presents the output characteristics of both devices, highlighting the superior current-driving capability of the S^3^-FinFET across a range of gate voltages. This enhancement is attributed to the improved electrostatic control enabled by the high-k stepped spacer, which strengthens gate-to-channel coupling.

**Figure 5:**
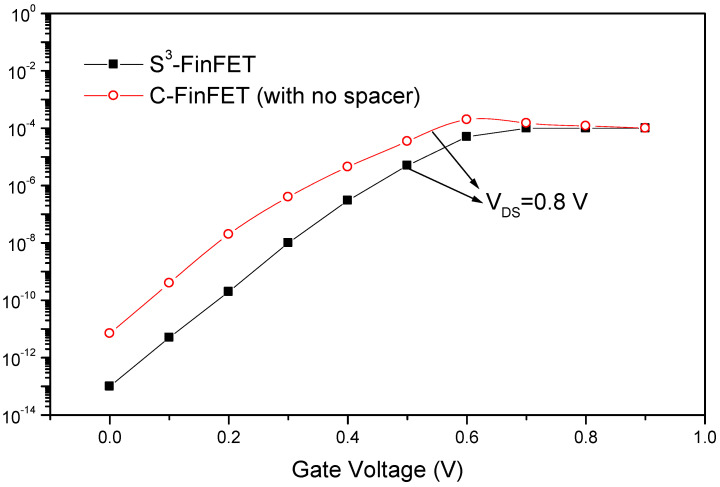
Transfer characteristics of S^3^-FinFET and conventional FinFET.

Additionally, Figure 5 shows the transfer characteristics of both devices. It can be seen that the S^3^-FinFET exhibits a significantly higher I_ON_/I_OFF_ ratio, confirming the effectiveness of the proposed spacer in enhancing device performance. These comparisons provide clear evidence that the S^3^-FinFET structure offers substantial benefits over conventional designs in terms of both current driving and short-channel control.

**Figure 6:**
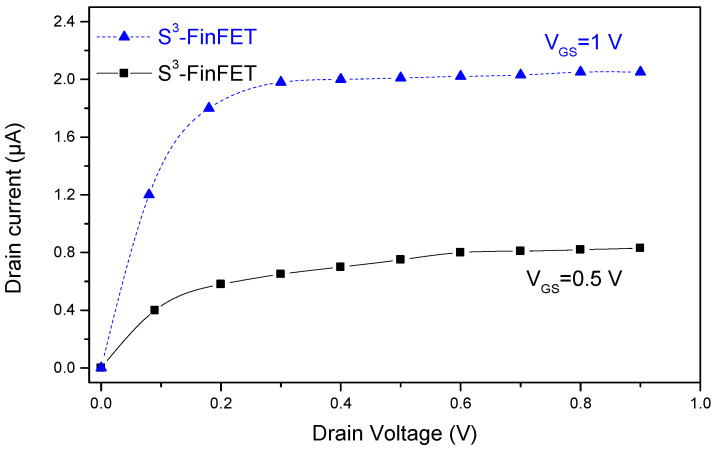
Output characteristics (I_D_–V_DS_) of the proposed S^3^-FinFET at V_GS_ = 0.5 V and V_GS_ = 1 V.

To evaluate the current-driving capability and short-channel behavior of the proposed S^3^-FinFET under different biasing conditions, output characteristics were analyzed at two gate voltages, V_GS_ = 0.5 V and V_GS_ = 1 V, as shown in Figure 6. In the figure, the curves exhibit a clear transition from the linear region to the saturation region, reflecting strong channel modulation and proper saturation behavior. At V_GS_ = 0.5 V, the drain current increases linearly with V_D_ in the low-voltage region, indicating good channel formation, while saturation occurs at higher V_D_ values, demonstrating effective gate control. At V_GS_ = 1 V, a significantly higher drain current is observed across all V_D_ values, highlighting the structure’s ability to support strong inversion and enhanced carrier injection due to improved electrostatics. The stepped high-k spacer in the S^3^-FinFET enhances fringe field coupling and reinforces gate control across the fin body, minimizing channel length modulation even at scaled dimensions. The saturation region shows minimal slope, which further confirms the suppression of drain-induced barrier lowering (DIBL). The nearly ideal output behavior at both gate voltages supports the effectiveness of the proposed design in maintaining high drive current and strong saturation, making it well-suited for ultra-scaled low-power applications.

**Figure 7:**
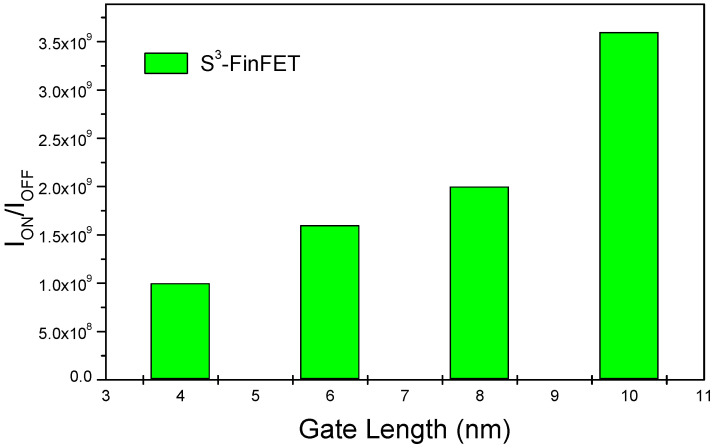
I_ON_/I_OFF_ ratio values of the S^3^-FinFET, showing peak performance exceeding 10^8^ due to low leakage and efficient carrier injection.

Figure 7 presents I_ON_/I_OFF_ ratio values of the proposed S^3^-FinFET. In the figure, a peak value exceeding 10^8^ can be seen. This high ratio reflects the device’s excellent ability to combine low leakage with strong drive current. The OFF-state current remains impressively low, in the order of ~10^−13^ A/μm, primarily due to the junctionless design and the well-engineered potential barrier within the channel. Uniform doping prevents abrupt junction transitions that typically act as leakage paths, resulting in smooth conduction band profiles and suppressed leakage in the OFF state. Moreover, the stepped spacer structure effectively reduces source-to-drain tunnelling by decoupling the drain electric field from the source/channel interface, maintaining a robust potential barrier under reverse bias. At the same time, the high ON current is enabled by efficient carrier injection from the source into the heavily doped channel, facilitated by the absence of depletion zones typical of junction-based devices. Additionally, quantum confinement within the narrow fin slightly increases the effective bandgap, further reducing OFF-state leakage and enhancing overall device switching performance.

**Figure 8:**
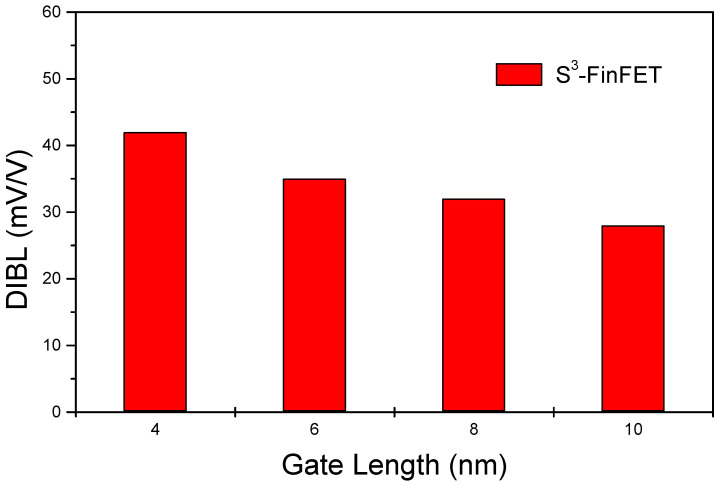
DIBL versus gate length for the S^3^-FinFET.

**Figure 9:**
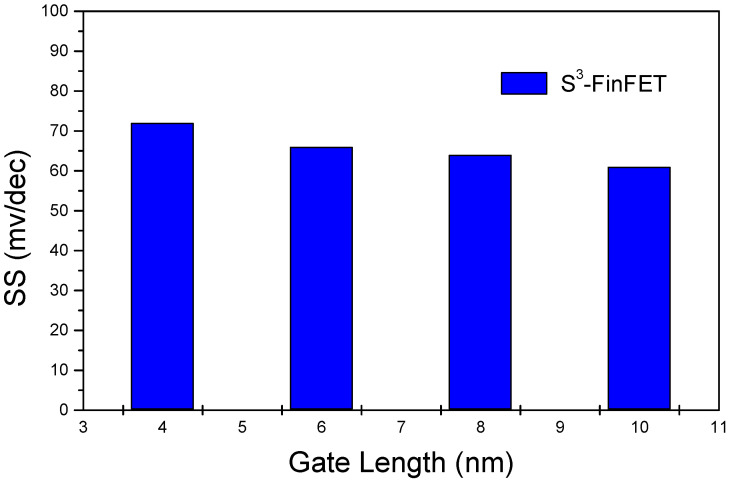
Subthreshold swing (SS) versus gate length for the S^3^-FinFET, demonstrating stable and low SS enabled by SOI isolation.

The DIBL and subthreshold swing (SS) of the proposed S^3^-FinFET are plotted in Figure 8 and Figure 9, respectively. DIBL is calculated by measuring the change in threshold voltage (V_th_) with respect to the change in drain voltage (V_D_), using the following formula [20]:

(2) $$DIBL=\frac{V_{th(V_{D}=low)}-V_{th(V_{D}=high)}}{V_{D,high}-V_{D,low}}$$

where Vth_(VD=low)_ and Vth_(VD=high)_ are the threshold voltages extracted at low and high drain biases, respectively. This parameter quantifies the extent to which the drain voltage influences the channel barrier, indicating short-channel effects.

The DIBL remains below 50 mV/V across the entire simulated gate length range, reflecting excellent suppression of drain-induced barrier lowering in the S^3^-FinFET. This performance is attributed to the serial capacitance effect of the three-layer spacer stack. Specifically, the low-k Si_3_N_4_ middle layer acts as a bottleneck that impedes the penetration of the drain’s electric field into the channel. As a result, a significant portion of the drain-induced voltage drops across the spacer itself, minimizing potential modulation of the channel barrier and enhancing threshold voltage stability under bias stress.

Meanwhile, the subthreshold swing exhibits minimal variation with changes in gate length and fin width, owing to the strong electrostatic insulation provided by the silicon-on-insulator (SOI) substrate and buried oxide (BOX) layer. These layers effectively block substrate-related parasitic effects, leading to consistent and improved subthreshold characteristics.

**Figure 10:**
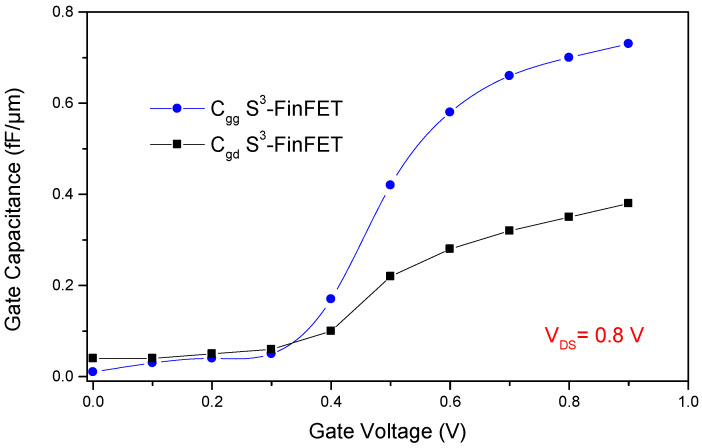
Gate-to-drain capacitance (C_gd_) and total gate capacitance (C_gg_) of the S^3^-FinFET versus V_GS_.

**Figure 11:**
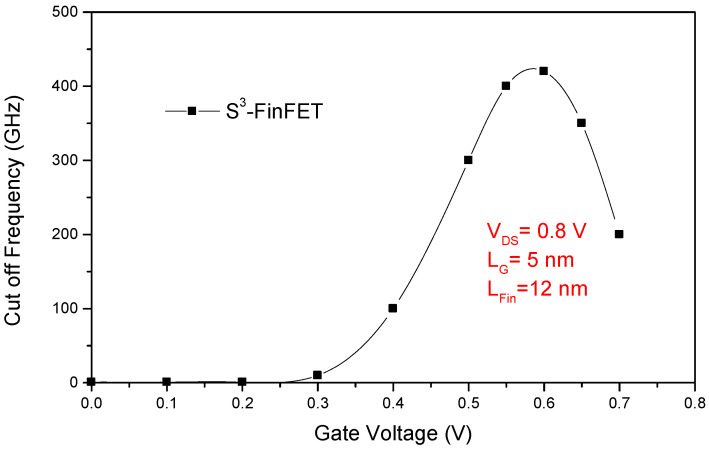
Cut-off frequency (f_T_) of the S^3^-FinFET versus V_GS_, highlighting high-speed performance enabled by optimized gate and spacer design.

Figure 10 illustrates the gate-to-drain capacitance (C_gd_) and total gate capacitance (C_gg_) of the proposed S^3^-FinFET as functions of the gate voltage (V_GS_), while the cut-off frequency (f_T_) versus V_GS_ is presented in Figure 11. The relatively low C_gd_, commonly known as the Miller capacitance or feedback capacitance, plays a crucial role in minimizing delay and dynamic power consumption, especially in FPGA interconnects and logic switches where fast and efficient signal transitions are essential. The S^3^-FinFET exhibits good behavior in terms of gate-to-drain capacitance. Additionally, the total gate capacitance, C_gg_, is the sum of C_gs_ and C_gd_, reflecting both the intrinsic gate-channel coupling and parasitic capacitances. The near-linear variation of C_gg_ with V_GS_ suggests stable gate modulation with minimal charge trapping effects across the operating voltage range, indicating reliable device behavior under different bias conditions.

The cut-off frequency (f_T_), calculated using a small-signal approximation, captures the high-frequency performance of the device. This figure of merit benefits from the combination of high transconductance and relatively low total gate capacitance, both resulting from the optimized gate electrostatics and spacer engineering in the S^3^-FinFET. Efficient coupling between the gate and the channel reduces capacitive delays, enabling the device to sustain high-speed operation, which is critical for RF and high-frequency digital applications.

Negative Bias Temperature Instability (NBTI) is a key reliability concern in transistors [21]. NBTI occurs when a device is subjected to negative gate bias (V_G_ < 0) at elevated temperatures, which is typical during circuit operation. Over time, this stress causes the generation of interface traps and positive fixed charges in the gate dielectric near the interface. These defects lead to a shift in the threshold voltage (ΔV_th_), reducing drive current and degrading device performance. As device dimensions scale, quantum confinement pushes the inversion-layer charge centroid away from the interface [21]. Therefore, understanding and mitigating NBTI in a device’s structure is critical for ensuring long-term device reliability.

**Figure 12:**
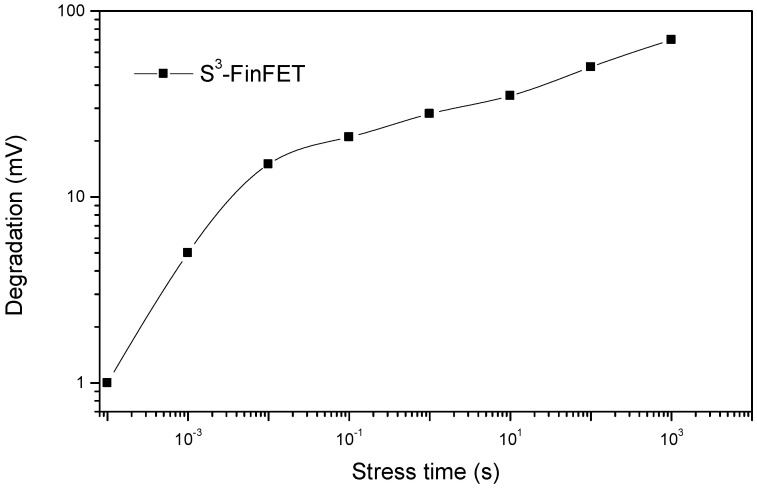
NBTI-induced threshold voltage shift during stress.

**Figure 13:**
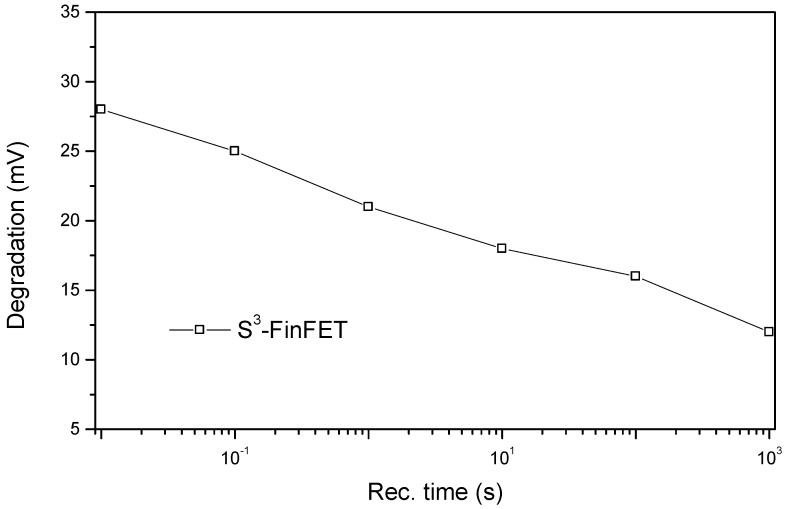
Threshold voltage recovery after removal of NBTI stress.

In this study, NBTI stress is simulated using Silvaco by applying a negative gate bias to the channel device to replicate the stress conditions responsible for NBTI degradation. Interface trap generation and recovery behavior are approximated using defined trap parameters at the Si/SiO_2_ interface within the simulation environment. Transient simulations are employed to observe the evolution of threshold voltage shift (ΔV_th_) during both stress and recovery intervals. This method enables an estimation of NBTI effects on device reliability under realistic operating conditions. The results are shown in Figure 12 and Figure 13. During NBTI stress, the threshold voltage shift (ΔV_th_) increases over time, mainly due to the continuous growth of interface traps. This causes ΔV_th_ to worsen as stress time progresses. When the stress is stopped and recovery begins, ΔV_th_ decreases gradually because of trap passivation and neutralization, but this recovery happens over a much longer time scale compared to the fast initial increase during stress.

**Figure 14:**
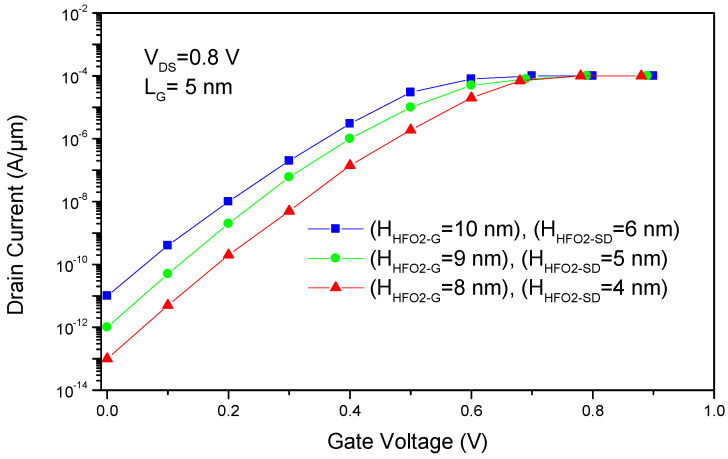
Spacer-height dependence of electrical performance in the S^3^-FinFET, showing the trade-off between leakage suppression and electrostatic control.

**Figure 15:**
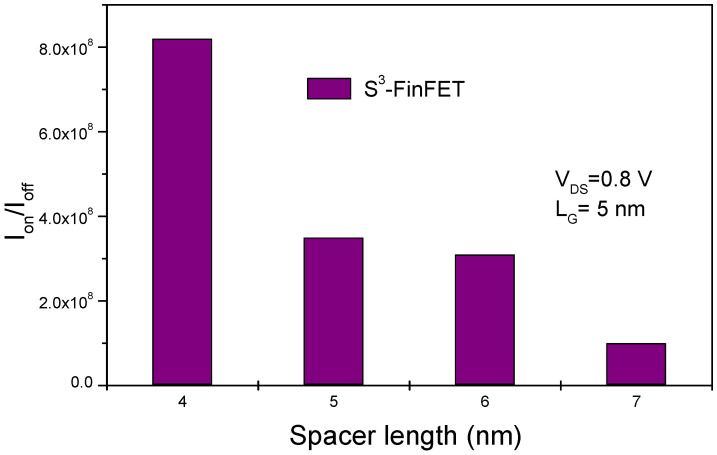
Impact of spacer length reduction on the I_ON_/I_OFF_ ratio, demonstrating improved gate control with optimal scaling.

The impacts of spacer height and length scaling are critical factors influencing the electrical performance of the proposed S^3^-FinFET structure. Figure 14 and Figure 15 show the effects of variations in spacer height and spacer length, respectively.

Increasing the spacer height in the proposed S^3^-FinFET, while keeping gate and spacer length fixed, reduces the gate’s ability to effectively control the channel. This weaker control allows more leakage current to flow when the transistor should be off, decreasing power efficiency. Additionally, as spacers’ heights increase, the aspect ratio of the spacer’s height to its length decreases, meaning that the spacer becomes relatively thinner compared to its height. This change in device geometry further contributes to increased leakage currents and degraded overall device performance.

Decreasing the spacer length brings the source and drain regions closer to the gate, which can improve short-channel control and enhance the I_ON_/I_OFF_ ratio. This happens because a narrower spacer reduces the distance between the gate and the lightly doped drain extension regions, allowing the gate to better suppress leakage and enhance drive current. However, too much reduction in spacer length can lead to parasitic capacitance increase and short-channel effects, which may eventually impair performance. Moderate spacer length scaling can therefore increase I_ON_/I_OFF_.

To evaluate the advantages of the proposed S^3^-FinFET structure, a performance comparison was conducted with another configuration: a FinFET using only HfO_2_ as the spacer material. Key device parameters, including I_ON_/I_OFF_ ratio, drain-induced barrier lowering (DIBL), and subthreshold swing (SS) are summarized in Table 2.

The S^3^-FinFET achieves a significantly higher I_ON_/I_OFF_ ratio, indicating that drive current is improved while low off-state leakage is maintained. Furthermore, the S^3^-FinFET exhibits markedly reduced DIBL and SS values, suggesting better electrostatic control and reduced short-channel effects. These results confirm that the S^3^ spacer configuration offers superior device performance compared to the FinFET with HfO_2_-only spacer.

## 4. Conclusions

This work presents the S^3^-FinFET, a novel junctionless SOI FinFET architecture featuring a stepped high-k three-layer spacer structure. The proposed design significantly enhances gate control and suppresses short-channel effects through improved fringe field coupling and electric field screening. TCAD simulation results demonstrate superior performance in terms of subthreshold swing, DIBL, ON/OFF current ratio, and high-frequency response when compared to conventional FinFET structures. The low Miller capacitance and high cut-off frequency make the device particularly attractive for FPGA applications, where reduced leakage and high-speed operation are essential. The compact and manufacturable design of the S^3^-FinFET makes it a promising candidate for energy-efficient, ultra-scaled logic components in next-generation reconfigurable systems.

## Figures and Tables

**Table 1:** Device parameters of the S^3^-FinFET used in TCAD simulations.

Parameter	Value
Gate length (LG)	5 nm
Fin length	12 nm
Fin thickness (t_Fin_)	8 nm
Gate oxide thickness	0.75 nm
HfO_2_ spacer height in gate side (H_HFO2-G_)	8 nm
HfO_2_ spacer height in source/drain side (H_HFO2-SD_)	4 nm
Si_3_N_4_ height	6 nm
Spacer length	4 nm
Source/drain doping	1 × 10^19^ cm^−3^
Source/drain length	12 nm
Buried oxide thickness	80 nm

**Table 2:** Comparison of performance metrics between S^3^-FinFET and FinFET with HfO_2_-only spacer.

Structure	I_ON_/I_OFF_	DIBL (mV/V)	SS (mV/dec)
S^3^-FinFET	1.41 × 10^9^	36.2	66.3
FinFET with only HfO_2_ spacer	9.34 × 10^8^	52.7	72.1

## Data Availability

Data is contained within the article.
